# Inflammasome Targeted Therapy in Pregnancy: New Insights From an Analysis of Real-World Data From the FAERS Database and a Systematic Review

**DOI:** 10.3389/fphar.2020.612259

**Published:** 2021-01-20

**Authors:** Carla Carnovale, Enrico Tombetti, Vera Battini, Faizan Mazhar, Sonia Radice, Mariangela Nivuori, Enrica Negro, Silvia Tamanini, Antonio Brucato

**Affiliations:** ^1^ Unit of Clinical Pharmacology, Department of Biomedical and Clinical Sciences L. Sacco, "Luigi Sacco" University Hospital, Università di Milano, Milan, Italy; ^2^ Department of Biomedical and Clinical Sciences, Università di Milano, Fatebenefratelli Hospital, Milan, Italy; ^3^ Internal Medicine, Fatebefratelli Hospital, Milan, Italy

**Keywords:** pregnancy, pharmacovigilance, inflammasome targeted therapy, IL-inhibitors, colchicine, pharmacovgilance

## Abstract

The published experience with biologics in childbearing age with autoimmune and inflammatory diseases mainly deals with the use of TNFα inhibitors (TNFα-i). Limited data are available for biologics targeting other cytokines or immunocompetent cells, especially for the inflammasome targeted therapy including IL-1 inhibitors and colchicine. We conducted a nested case-control study by using the US Food and Drug Administration Adverse Event Reporting System database aimed at quantifying the association between the use of IL-1 inhibitors/colchicine in pregnant women and the occurrence of maternal/fetal adverse effects. The reporting odds ratio was used as a measure of disproportional reporting. From the total cohort (40,033 pregnant women), we retrieved 7,620 reports related to neonatal AEs, 2,889 to fetal disorders, 8,364 to abortion, 8,787 to congenital disorders, and 7,937 to labor/delivery complications. Inflammasome-targeted drugs did not present any disproportionate reporting for all these clusters of AEs. TNFα-i confirmed their safety during pregnancy with aROR < 1 for all clusters of AEs except for labor complications. Finally, we performed a systematic review of the current literature. Data from the eligible studies (12 observational studies and 6 case reports; yielding a total of 2,075 patients) were reassuring. We found no major safety issues on malformations risk of inflammasome targeted therapies in pregnancy. However, due to limited data, the routine use of these agents should be considered in pregnancy only if risk benefit assessment justifies the potential risk to the fetus.

## Introduction

In the past 2 decades, the number of women in childbearing age with autoimmune and inflammatory diseases has significantly increased, in parallel with some concerns on the safety of biological drugs used to treat these clinical conditions. A huge number of therapeutic options have become available, which may ensure optimal control of disease activity and markedly ameliorate patients’ quality of life. Despite the therapeutic armamentarium to manage pregnant women with systemic autoimmune rheumatic diseases (SARDs) includes a variety of biological agents, scant data are available concerning their use during reproduction and pregnancy, especially in long-term studies (Østensen et al., 2008; Østensen et al., 2015).

Biologics have firstly become available about 20 years ago. Since then, the published experience with biologics during pregnancy mainly deals with the use of TNFα inhibitors (TNFα-i) (Flint et al., 2016; Skorpen et al., 2016; Østensen, 2017); data from a growing number of publications on the use of TNFα-i pregnant women with most autoimmune diseases is reassuring (Smeele and Dolhain, 2019).

In contrast, pregnancy experience of exposure to other monoclonal antibodies targeting cytokines or immunocompetent including CD20 (rituximab), interleukin (IL)-6 receptors (tocilizumab), IL-17A (secukinumab) and nucleotide-binding domain (NOD)-like receptor protein 3 (NLRP3) inflammasome, has been limited to conference abstracts or simple case reports (Komaki et al., 2017). With regard to therapy of NLRP3-associated diseases, three biologic agents blocking IL-1 are available such as the neutralizing IL-1β antibody canakinumab, the recombinant IL-1 receptor antagonist anakinra, and the soluble decoy IL-1 receptor rilonacept (Dinarello and van der Meer, 2013; Zahid et al., 2019; Klein et al., 2020).

These agents have been used during pregnancy only recently, starting from mothers that required maintenance of biologic therapy to control the undelaying condition. However, the need of a prolonged drug administration during gestation raises many controversial issues about the effects of *in utero* exposure to these agents on the fetus/maternal health. Biologic agents are increasingly used and thus long-term data on the effect of these therapies on fetus development and maternal outcomes would be highly necessary. Prospective registries, some of which are already in place, are intended to serve at the purpose but their data are not available yet. Alternatively, spontaneous reporting systems represent a valuable source of information in frail populations, such as pregnant women. Despite the intrinsic limitations, data-mining of adverse drug reaction (ADR) reports allow to obtain real word data about the safety/efficacy profile of specific drugs, to compare therapeutic options, and gain insight on potential mechanisms of ADRs (Gentili et al., 2018; Perrotta et al., 2019).

In the past decades, no pharmacovigilance studies using spontaneous reporting system database specifically address the inflammasome targeted therapy potential risk of harm in pregnant women or the outcomes in their infants. We thus conducted a case-control study by using the US Food and Drug Administration Adverse Event Reporting System (FAERS) database aimed at quantifying the association between the use of inhibitors of the NLRP3 inflammasome in pregnant women and the occurrence of maternal and fetal adverse effects. Finally, in view of the growing number of studies on this issue, we performed a systematic review of the current literature to include and discuss real-world data, thus contributing to fill the knowledge gaps regarding safe and effective use of new biological drugs in pregnancy.

## Materials and Methods

### US Food and Drug Administration Adverse Event Reporting System Analysis: Data Source and Study Design

In order to appropriately estimate the incidence of pregnancy and neonatal adverse events (AEs) after the administration of inflammasome-targeted drugs, an analysis of the FAERS^®^ has been conducted. FAERS^®^ is an online database maintained by FDA that collects every adverse drug reaction (ADR) report submitted in the US territory and every serious ADR report filed in all 150 countries enrolled in the Program for International Drug Monitoring (PIDM) established by the World Health Organization (WHO).

In FAERS^®^, healthcare professionals, patients and marketing authorization holders may submit ADR reports in the form of Individual Case Safety Reports (ICSRs). FAERS^®^ employs the latest version of the Medical Dictionary for Regulatory Activities (MedDRA^®^) in order to properly encode every ADR and WHO Anatomical Therapeutic Chemical (ATC) codes to standardize drug nomenclature.

ICSRs provide administrative information (country, type of report, qualification of the reporter), patient demographics (gender, age, weight), adverse events (seriousness of the ADR, date of onset reaction, outcome), information about drug therapy (drug name, drug start and stop dates, time to onset, dose, indication, dechallenge and rechallenge), but the level of completeness of information varies from case to case (Sakaeda et al., 2013). As the number of safety reports sent to the FDA annually is continuously expanding, the database is largely used to detect novel drug-related safety events (Carnovale et al., 2019a; Carnovale et al., 2019b; Mazhar et al., 2019; Pozzi et al., 2019).

#### Data Acquisition and Data Processing

This study was designed as a nested case-control study and data were downloaded from the FAERS Public Dashboard (FDA Public Dashboard, 2020). The base cohort consisted of all cases involving any AEs occurred during pregnancy (search terms: “complication of pregnancy”; “Exposure during pregnancy”; “Fetal exposure during pregnancy”; “Maternal exposure during pregnancy”).

The study period covered the first quarter of 2010 to the first quarter of 2020.

Duplicate records were detected and deleted accordingly as previously described (Carnovale et al., 2019; Mazhar et al., 2019). In order to reduce the risk of a misleading relationship between drugs and AEs, only reports entered by healthcare professionals were eligible for the final cohort.

#### Definition of Cases and Controls

Cases were defined as all Individual Case Safety Reports (ICSRs) where inflammasome-targeted drugs were reported as suspect drug. We then used the Standardized MedDRA Queries (SMQ) related to the group “Pregnancy and neonatal topics” to identify the risk of particular clusters of AEs in our cases. To find ICSRs of interest (*i.e.,* cases reporting any maternal and/or fetal outcome following the use anti-IL drugs/colchicine) in FAERS database, we used the following SMQs: congenital, familial and genetic disorders; fetal disorders; neonatal disorders; termination of pregnancy and risk of abortion; pregnancy, labor and delivery complications and risk factors (excl. abortions and stillbirth) (Introductory Guide for Standardised MedDRA Queries, 2020).

#### Statistical Analyses

Descriptive analysis was performed for cases and non-cases, in terms of age, male sex (new-borns’ ICSR) and the presence of other co-suspect drugs.

To identify signals of disproportionate reporting (SDR) for pregnancy and neonatal outcomes in association with inflammasome-targeted drugs, we used the reporting odds ratio (ROR) as a measure of disproportional reporting, which estimates the frequency of an event of interest with the tested drugs compared with the other drugs. SDR were detected when the number of reports was higher than three and ROR–95% CI was greater than one. We calculated the adjusted ROR (aROR) by using multivariate logistic regression analysis adjusted for the presence of co-suspects, that was considered a potential confounding factor.

Sub-analysis was then performed to compare anti-IL1 agents and colchicine with TNFα-i, a well-known class of drugs used during pregnancy chronically and safely. We used R software for data tiding and statistical analysis (R core team, 2019).

### Systematic Review

#### Literature Search

We followed the Preferred Reporting Items for Systematic Reviews and Meta-analysis (PRISMA) guidelines (Moher et al., 2009) (Supplementary Table S1). We searched PubMed/MEDLINE, EMBASE databases up to June 23rd, 2020 with no language restriction. We provided our search strategy for PubMed in the Supplementary material (Supplementary Material S1); the search strategy was adapted as needed for each database. In brief, we used two terms: Interleukin 1 Receptor Antagonist/colchicine, and Pregnancy. We combined terms with the Boolean operator “AND”.

#### Eligibility Criteria

Inclusion criteria for the qualitative analysis were: studies enrolling human subjects; studies enrolling pregnant women affected by any clinical conditions treated with any anti-IL drugs/colchicine; studies reporting any maternal and/or fetal outcome following the use anti-IL drugs/colchicine; any type of observational studies and clinical trials; case report/case series.

We excluded: literature reviews; conference proceedings/abstracts and unpublished thesis; studies that did not report outcomes of interest; studies that reported the effect of pregnancy on IL-1 Inhibitor therapies/colchicine rather than vice versa.

We identified additional articles potentially eligible through the reference lists of articles included in our systematic review. We did not contact authors for missing data.

#### Study Selection

We imported into EndNote all the retrieved references from the final search in order to eliminate duplicate records. We screened the titles and abstracts of potentially eligible papers; whereas, we disregarded the papers deemed highly unlikely to be relevant. Finally, we assessed for eligibility all full-text versions of the remaining articles based on our pre-specified eligibility criteria as previously described. Two independent reviewers (CC and VB) conducted the entire search process. Discrepancies between review authors were resolved by discussion with a third review author (ET) to achieve a decision.

#### Data Extraction and Synthesis

Data from all included studies were extracted using pre-specified forms. The following relevant information was extracted: study type, study duration, number of subjects, number of women treated with biological agents, number of pregnancies, maternal diagnosis, mean age at conception, drug of interest, dose/scheme, concomitant treatment, any maternal and/or fetal outcome following the use of Interleukin 1 Receptor Antagonists/colchicine (including live birth, miscarriages, early/late/voluntary abortion, fetal death, small for gestation age-SGA, congenital malformation and stillbirth) in pregnant women, type of delivery, week at delivery, APGAR score, and duration of follow-up.

## Results

### Pharmacovigilance Study

#### Study Population

Of 11,789,929 ICSRs we retrieved from the FAERS in our period of observation, 40,033 involved maternal, fetal and neonatal reactions. Of these, only 84 (0.2%) reported the drugs of interest as “suspect,” i.e., 69 for Anti-IL1 and 15 for colchicine; no reports involved both drugs as “suspect.” The mean age between cases and non-cases was similar in the pregnant women (29 ± 6 vs. 31 ± 7 years old) with a range between 15 and 68 years old. Among the 84 cases, 24 ICSRs reported AEs for newborns and the maternal diagnoses mostly reported were “Familial Mediterranean Fever” (11 ICSRs) and “Rheumatological diseases” (14 ICSRs). In 59.5% of cases, more than one suspect drug was reported; 48.5% of non-cases reported co-suspect drugs.

#### Disproportionality Analysis

The number of cases, the crude and adjusted ROR for each cluster of AEs are presented in Table 1. From the total cohort, we retrieved 7,620 reports related to neonatal AEs, 2,889 to fetal disorders, 8,364 to abortion, 8,787 to congenital disorders, and 7,937 to labor/delivery complications.

**TABLE 1:** Disproportionality analysis of adverse drug reactions spontaneous reporting database FAERS for the identification of Pregnancy and neonatal disorders.

	Cases (n)^a^	Non cases (n)^b^	ROR (95% CI)	aROR (95% CI)^c^
Neonatal disorders (n = 7,620)				
Inflammosome targeted	18	7,602	1.16 (0.67; 1.91)	1.09 (0.62; 1.80)
Anti-IL1	14	7,606	1.08 (0.58; 1.89)	1.06 (0.56; 1.86)
Colchicine	4	7,616	1.55 (0.43; 4.53)	1.20 (0.33; 3.50)
Anti-TNFα	487	7,133	0.67 (0.61; 0.74)^d^	0.74 (0.67; 0.82)^d^
Fetal disorders (n = 2,889)				
Inflammosome targeted	7	2,882	1.17 (0.49; 2.36)	1.11 (0.47; 2.25)
Anti-IL1	6	2,883	1.23 (0.47; 2.61)	1.11 (0.47; 2.25)
Colchicine	1	2,888	—	—
Anti-TNFα	185	2,704	0.71 (0.60; 0.82)^d^	0.76 (0.65; 0.88)**
Labor/delivery complications (n = 7,937)				
Inflammosome targeted	19	7,918	1.18 (0.69; 1.93)	1.18 (0.69; 1.93)
Anti-IL1	17	7,920	1.32 (0.74; 2.23)	1.32 (0.74; 2.24)
Colchicine	2	7,935	—	—
Anti-TNFα	852	7,085	**1.36 (1.25;1.47)** **^d^**	**1.36 (1.25;1.47)** **^d^**
Abortion (n = 8,364)				
Inflammosome targeted	19	8,345	1.11 (0.65; 1.80)	1.17 (0.68; 1.91)
Anti-IL1	17	8,347	1.24 (0.70; 2.10)	1.26 (0.71; 2.14)
Colchicine	2	8,362	—	—
Anti-TNFα	770	7,594	1.09 (1.00; 1.18)^e^	1.01 (0.93; 1.10)
Congenital disorders (n = 8,787)				
Inflammosome targeted	20	8,767	1.11 (0.66; 1.80)	1.03 (0.61; 1.68)
Anti-IL1	14	8,773	0.91 (0.48; 1.58)	0.88 (0.47; 1.55)
Colchicine	6	8,781	2.37 (0.80; 6.58)	1.78 (0.60; 4.96)
Anti-TNFα	323	8,464	0.34 (0.30; 0.38)^d^	0.38 (0.33; 0.42)^d^

^a^ All Individual Case Safety Reports where the use of inflammasome-targeted therapy/Anti-TNFα drugs during pregnancy were reported as suspect in the occurrence of any maternal/neonatal/fetal disorders.

^b^ All Individual Case Safety Reports where the use of inflammasome-targeted therapy/Anti-TNFα drugs during pregnancy were reported as suspect drug in the occurrence of any adverse events.

^c^ Adjustment for other co-suspects.

^d^ *p* < 0.001.

^e^ *p* < 0.01.

In bold: aROR >1.

According to the aROR and 95% CI, inflammasome-targeted drugs did not present any disproportionate reporting for all these clusters of AEs. Next, we performed a sub-analysis for individual drugs, including IL-1 inhibitors and colchicine and using TNFα-i as a better-characterized reference. No disproportionate reporting was identified for IL-1 inhibitors and colchicine. TNFα-inhibitors confirmed their safety during pregnancy with aROR <1 for all clusters of AEs except for labor complications.

### Systematic Review

#### Characteristics of the Reviewed Studies

**FIGURE 1:**
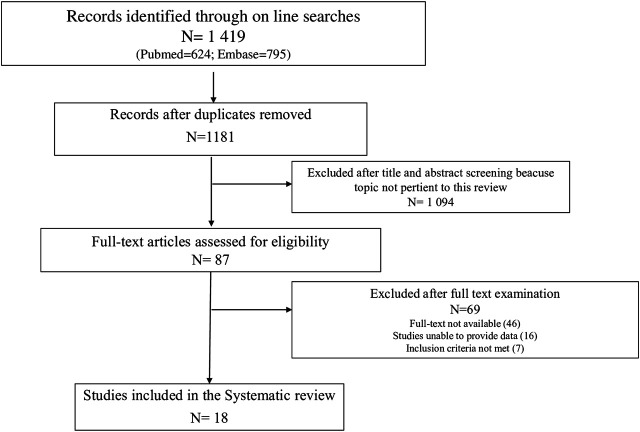
PRISMA (Preferred Reporting Items for Systematic Reviews and Meta-analyses) flow diagram of process of study selection.

The study selection and screening are presented in the PRISMA flow-chart (Figure 1). Of the 1,419 articles retrieved (624 results were from PubMed, and 795 from EMBASE), 18 met the inclusion criteria.

Twelve observational studies and 6 case report/series were eligible for the inclusion in our systematic review (Table 2), yielding a total of 2,075 patients. No clinical trials reporting maternal and/or fetal outcome following the use of Interleukin 1 Receptor Antagonists/colchicine in pregnant women were found.

**TABLE 2:** Detailed overview of published data on Inflammosome target therapies and evidence for their use during pregnancy.

Id	Study design	Maternal diagnosis	Pregnant treated with drugs of interest/total cohort (no. total pregnancies)	Dose	Mean age at conception (years)	Weeks of gestation; no. of pregnancies: delivery type	APGAR Score	No. Pregnancy outcome in women treated with drugs of interest/control group	Additional relevant data	Follow-up
Brucato et al. (2019)	Retrospective/prospective cohort	IRP	2/14 (21)	Colchicine 1 mg 5 days/week during all the pregnancy	25	At week 40	nr	2: good maternal outcome	na	1 year after delivery
				Colchicine 1 mg/daily during all the pregnancy	36	At week 38		2: live birth		
Ben-Chetrit et al. (2010)	Case control	FMF	*Group 1:* 61/132 (179)	Colchicine 1.0–1.5 mg daily (throughout all pregnancies)	24.4 ± 3.7^a^	17: CS	nr	16: early abortions	The number of early and late abortions, congenital malformations were higher in groups 2 and 3 compared with group 1 but did not reach the level of statistical significance	10 years
								2: late abortions		
								1: VSD/ASD		
			*Group 2:* 22/132 (104)	Colchicine 1.0–1.5 mg daily (during some pregnancies)	23 ± 4.3	5:CS	nr	23: early abortions		
								3: late abortions		
								1: died		
								1: ccenter lip		
								1: hydronephrosis		
			*Group 3:* 49/132 (197)	Colchicine 1.0–1.5 mg daily (no during pregnancies)	23.2 ± 4.8	18: CS	nr	27: early abortions		
								4: late abortions		
								1: Fallot’s		
								1: ccenter lip		
		Healthy women	*Control group:* 84 (312)	na	40.8 ± 11	nr	nr	46: early abortions		
								6: late abortions		
								2: major congenital malformations		
								1: mild muscle weakness		
Bodur et al. (2020)	Cross-sectional	FMF	*Group 1:* 62/401 (585)	Colchicine nr (during pregnancy)	na	nr	nr	12: pre-term delivery	Pregnant/neonatal disorders were similar among FMF patients regardless of colchicine use	nr
								13: abortus		
								8: stillbirth		
								1: fetal anomaly		
			*Group 2:* 339/401 (585)	Colchicine free	na	nr	nr	29: pre-term delivery		
								93: abortus		
								24: stillbirth		
								9: fetal anomaly		
Dağlar et al. (2016)	Prospective case control	FMF	*Group 1:* 33 pregnancies	Colchicine 1.5–2 g	25 ± 4.63^b^	21: VD	3: Apgar ≤7	7: preterm delivery	No significant differences in preterm delivery (*p* = 0.73), preeclampsia (*p* = 1) low Apgar scores (*p* = 1) and NICU admission (*p* = 1) between the groups. No cases of fetal chromosomal anomaly or perinatal mortality in either of the groups	From the first trimester to the end of the pregnancies
						12: CS		1: birth weight <10th percentile		
								4: NICU admission		
		Healthy pregnant	*Control group 2:* 32 pregnancies	na	28.5 ± 7.2^c^	21: VD	4: Apgar ≤7	1: preeclampsia		
						11: CS		7: preterm delivery		
								1: birth weight <10th percentile		
								3: NICU admission		
Diav-Citrin et al. (2010)	Prospective comparative cohort	FMF (87.3%) Bechet disease (7.5%) or other (5.2%)	*Group 1:* 238 pregnancies	Colchicine 1 mg daily (97%: at least in the first trimester)	29^b^ (median)	nr	nr	12: miscarriage	The rate of major anomalies, after first-trimester exposure was comparable between the 2 groups *p* = 0 0.288	nr
								10: major anomalies^c^		
								32: preterm delivery		
		nr	*Group 2:* 964 pregnancies	Counseled for non-teratogenic exposure	30^b^ (median)	nr	nr	55: miscarriage		
								35: major anomalies		
								51: preterm delivery		
Ehrenfeld et al. (1987)	Prospective cohort	FMF	*Group 1:* 10/23 (28)	Colchicine (throughout the entire period of pregnancy)	nr	nr	nr	2: miscarriage	na	Upto 12 years
								9: live birth		
			*Group 2:* 17/25 (24)	Colchicine discontinued before attempting to conceive	nr	nr	nr	2: miscarriage	na	
								22: live birth		
Iskender et al. (2020)	Prospective case-control	FMF	Group 1: 55/66 (nr)	Colchicine (during pregnancy)	26.9 ± 5.6^b^	17: CS	nr	3: preeclampsia	Rates of preeclampsia, preterm delivery, SGA neonates and primary caesarean: similar between groups	nr
								8: SGA		
								9: preterm delivery		
								3: fetal anomaly^d^		
		Healthy women	Group 2: 11/66 (nr)	Patients non using colchicine	29.2 ± 6.2^b^	4: CS	nr	1: SGA		
								1: preterm delivery		
Orabona et al. (2020)	Case report	IRP	1 pregnancy	Colchicine 1.5 mg daily (during pregnancy)	30^b^	CS at 38/40 weeks	nr	1: good maternal outcome	Healthy male baby of adequate weight	nr
								1: live birth		
Tutuncu et al. (2006)	Case report	FMF	1 pregnancy	Colchicine 1.5 mg daily (throughout pregnancy)	20^b^	CS at 38/40 weeks	nr	1: good maternal outcome	Healthy female newborn without congenital anomaly	nr
								1: live birth		
Yazicioğlu et al. (2014)	Retrospective cohort	FMF	42/50 (50)	Colchicine	29.5 ± 5.1^b^	36.1 ± 4.0 weeks	nr	1: thanatophoric dysplasia	Preterm delivery (*p* = 0.058), low birth weight (*p* = 0.416) and congenital anomalies (*p* = 0.451) were not significantly associated with colchicine therapy	Until delivery
			8/50 (50)	Colchicine free		37.8 ± 1.7 weeks	nr	1: open type neural tube defect		
Bazzani et al. (2015)	Prospective cohort	Rheumatological diseases	1/67 (79)	Anakinra nr (during peri-conception period and/or during gestation)	33 ± 5	na	na	1: voluntary abortion	na	6 months after pregnancy
Berger et al. (2009)	Case report	AOSD	1 pregnancy	Anakinra 100 mg daily (2 years prior to and through pregnancy and delivery)	33^b^	VD at 41/40 weeks	7,8,9	1: live birth	This is the first report of treatment of AOSD with anakinra during pregnancy	4 months
								1: good maternal outcome		
Fischer-Betz et al (2011)	Case series	AOSD	Case 1: 1 pregnancy	Anakinra 100 mg daily (1 year prior to pregnancy and throughout)	27	VD at 39/40 weeks	9/9/10	1: live birth 1: good maternal outcome	There were no pathological findings on pediatric examination at discharge	nr

			Case 2: 1 pregnancy	Anakinra 100 mg daily (12/40 onwards)	29	CS at 36/40 weeks	8/9/9	1: live birth	nr	nr
								1: good maternal outcome		
Chang et al. (2014)	Prospective cohort	CAPS	6/9 (24)	Anakinra range: 100–300 mg daily (during pregnancy)	26.4 (range: 19–35)^b^	7: VD 2: CS	nr	1: miscarriage	nr	nr
								1: fetal demise of 1 fetus with renal agenesis in twin dichorionic-diamniotic pregnancy		
İlgen and Küçükşahin (2017)	Case report	FMF	1 pregnancy	Anakinra 100 mg daily (during pregnancy)	27	CS at 38/40 weeks	9,10	1: live birth	nr	13 months
								1: good maternal outcome		
Smith and Chambers (2018)	Prospective cohort	AOSD (60%)	5 pregnancies	Anakinra 100 mg daily (during pregnancy)	30.6 (range: 21.0–36.8)^b^	3: VD	nr	2: oligohydramnios^e^	Previous exposure throughout her pregnancy to celecoxib^e^	nr
		JIA				2: CS		3: jaundice		
		(40%)						1: tongue tied		
								1: right hydrocele, heart murmur resolved		
Youngstein et al. (2017)	Retrospective cohort	CAPS (66.6%)	7/7 (8)	Canakinumab (during pregnancy)	24 ± 5.1 (range: 16–32)	3: CS	Range: 9–10	1: miscarriage^§^	Both canakinumab and anakinra. Normal development^§^	Upto 10 years
		FMF (33.3%)				2: VD		7: live birth		
		Inflammatory illness (16.6%)				2: nr				
		CAPS (52.1%)	23/23 (23)	Anakinra (during pregnancy)	29 (range: 20–38)^b^	7: VD	Range: 7–10	1: miscarriage^§^	nr	
		AOSD (17.4%)				4: CS		1: ectopic neurohypophysis; center renal agenesis		
		FMF (13%)				2: ID		22: live birth		
		TRAPS (8.6%)								
		Pericarditis (4.3%)								
		Cogan Syndrome (4.3%)								
Egawa et al. (2017)	Case report	CAPS	1 pregnancy	Canakinumab 150 mg every 8 weeks (during pregnancy)	32	CS	8	1: live birth	First report of the administration of canakinumab during pregnancy in a CAPS patient	1 month after delivery

AOSD, adult-onset Still’s disease; CAPS, Cryopyrin Associated Periodic Syndrome; CS, cesarean section; FMF, Familiar Mediterranean Fever; ID, induced delivery; IRP, idiopathic recurrent pericarditis; IVF, *in vitro* fertilization; na, not applicable; MWS, muckle-Wells Syndrome; nr, not reported; SGA, Small for gestational age; VD, vaginal delivery; VSD/ASD, ventricular septal defect, atrial septal defect.

^a^ Age at first pregnancy.

^b^ Maternal age.

^c^ Muscular ventricular septal defect, duplex collecting kidney and supraventricular tachycardia.

^d^ Craniosynostosis; cerebral palsy; VSD; congenital hypothyroidism; urinary reflux; clubfoot; clubfeet; omphalocele; severe microcephaly.

^e^ Previous exposure throughout her pregnancy to celecoxib, a drug in the NSAID class that has been previously associated with the development of low amniotic fluid levels.

^§^ patient who received both canakinumab and anakinra in two separate pregnancies both resulting in miscarriage.

The average maternal age at conception ranges from 20 to 36 years. Of the 553 women treated with drugs of interest during pregnancy, 236 (42.6%) had a diagnosis of Familiar Mediterranean Fever (FMF); 3 (0.5%) of adult-onset Still’s disease (AOSD), 7 (1.2%) of Cryopyrin Associated Periodic Syndrome (CAPS), 3 (0.5%) of idiopathic recurrent pericarditis (IRP), and in the remaining cases a mixed cohort of women had various rheumatological diseases (FMF, CAPS, AOSD, Behcet’s disease, pericarditis, Cogan Syndrome, Juvenile idiopathic arthritis).

With respect to the type of medication used, 10 studies reported data on colchicine, 6 on anakinra, and 2 on canakinumab. The period from the baseline to the last follow-up varied considerably among the studies with a range from the first trimester to 10 years.

#### Data on the Use of anti-IL1 Drugs During Pregnancy

No studies reporting maternal and/or fetal outcome following the use of rilonacept were found.

Only a retrospective cohort study (Youngstein et al., 2017) and a case report (Egawa et al., 2017) provided data on the use of canakinumab during pregnancy (Table 2); in total, data were available for nine pregnancies; of these, eight completed pregnancies resulted in the normal development of the fetus; only 1 miscarriage in Cogan Syndrome patient was found.

Seven studies reported data on the use of anakinra in pregnant women (in total, data were available for 57 pregnancies).

In the prospective cohort study by Bazzani et al. (2015), only one woman took anakinra during a peri-conception period and gestation; the pregnancy resulted in a voluntary abortion. Data on case report/series (Fischer-Betz et al., 2011; Berger et al., 2009; İlgen and Küçükşahin, 2017) reported no pregnancy complications or fetal disorders in mothers with AOSD and FMF treated with anakinra (100 mg daily; 1–2 years prior to and through pregnancy and delivery). All pregnancies resulted in the normal development of a fetus.

Five pregnancies with anakinra exposure identified in the study by Smith and Chambers (2018) resulted in full-term singleton live births with no major or long-term complications. Two subjects developed oligohydramnios for unclear reasons.

Of twenty-three anakinra-exposed pregnancies in the retrospective study by Youngstein et al. (2017), 21 resulted in the birth of healthy infants (with follow-up of up to 10 years); one baby with unilateral renal agenesis and ectopic neurohypophysis was also reported. There were no serious neonatal infections.

In the prospective study by Chang et al. (2014) 6 women with CAPS were treated with anakinra throughout the pregnancy; no preterm births or serious pregnancy complications were reported and the miscarriage rates for pregnancies without and with anakinra treatment were 27% and 11%, respectively. One fetus of the twin pregnancy had renal agenesis and died *in utero*.

Despite reassuring findings, the lack of well-controlled studies make data on the use of anakinra in pregnancy difficult to interpret (Table 3).

**TABLE 3:** Findings of systematic reviews on anakinra and colchicine for each outcome included in the analysis.

OUTCOME	No. of cases in women exposed to Anakinra (% of total pregnancies^a^)	No. of cases in the control group (% of total pregnancies)	No. of cases in women exposed to Colchicine (% of total pregnancies^a^)	No. of cases in the control group (% of total pregnancies^b^)
Miscarriages	3 (5.3)	—	14 (1.2)	57 (2.9)
Abortions	—	—	31 (2.7)	145 (7.3)
Fetal/congenital anomaly	3 (5.3)	—	16 (1.4)	48 (2.4)
SGA	—	—	9 (0.8)	2 (0.1)
Stillbirth	—	—	8 (0.7)	24 (1.2)
Pre-term delivery	—	—	60 (5.2)	88 (4.5)
Voluntary abortion	1 (1.7)	—	—	—
Pre-eclampsia	—	—	3 (0.3)	1 (0.05)

^a^ Total of pregnancies in women treated with drugs of interest (n = 57 and n = 1,155, for anakinra and colchicine group, respectively). For two studies (Bodur et al., 2020; Yazicioğlu et al., 2014), the total number of pregnancies we considered includes women that did not receive the drug therapy; for the study by Iskender et al., as the total number of pregnancies was not available, we considered the total number of pregnant women treated with the drug of interest.

^b^ Total of pregnancies in the control group (n = 1978).

#### Data on the Use of Colchicine

Of 10 studies involving patients exposed to colchicine for any duration during pregnancy, eight were observational [of these, four were cohort studies (Ehrenfeld et al., 1987; Diav-Citrin et al., 2010; Yazicioğlu et al., 2014; Brucato et al., 2019); 3 case-control (Ben-Chetrit et al., 2010; Dağlar et al., 2016; Iskender et al., 2020); and 1 cross-sectional (Bodur et al., 2020)] and 2 were case report (Tutuncu et al., 2006; Orabona et al., 2020). Except for the study by Ehrenfeld *et al*, the remaining studies have been published recently (range: 2020–2006). Colchicine doses between 1 and 2 mg daily were reported. Two studies included healthy patients in their control groups (n = 116 women) (Ben-Chetrit et al., 2010; Dağlar et al., 2016).

In total, data were available for 1,155 pregnancies exposed to colchicine for any duration during pregnancy.

Of 1,152 pregnancies in women with FMF, 31% resulted in abortions, 14% in miscarriages, 16% in fetal/congenital anomalies, eight in stillbirth, 60 pre-term delivery; nine SGA and 3 preeclampsia.

There were three completed pregnancies in IRP patients, with good maternal/fetal outcomes (Orabona et al., 2020; Brucato et al., 2019). As reported in Table 3, findings from studies with control or comparator group reported no difference between the group of women treated with colchicine throughout all pregnancies and healthy women, in terms of miscarriages, early/late abortions, still-birth, fetal/congenital malformations, SGA, pre-term deliveries, and preeclampsia (Ehrenfeld et al., 1987; Ben-Chetrit et al., 2010; Diav-Citrin et al., 2010; Yazicioğlu et al., 2014; Dağlar et al., 2016; Iskender et al., 2020; Bodur et al., 2020). However, the robustness of uncontrolled evidence was poor in the reviewed studies.

Table 4 reported a summary of evidence on the use of Inflammasome target therapies in pregnancy.

**TABLE 4:** Brief summary of evidence on or Inflammosome target therapies use in pregnancy.

IL-1 Antagonist	Human data: *drug use throughout pregnancy*
Anakinra recombinant human IL-1 receptor antagonist	10 completed pregnancies in *AOSD* (Berger et al., 2009; Fischer-Betz et al., 2011; Youngstein et al., 2017; Smith and Chambers, 2018) →1 left renal agenesis; 2 jaundice and 1 tongue tied
	36 pregnancies in *CAPS* (Chang et al.,2014; Youngstein et al., 2017) → 1 fetal demise of 1 fetus with renal agenesis in twin pregnancy; 1 miscarriage
	4 completed pregnancies in *FMF* (Youngstein et al., 2017; İlgen and Küçükşahin, 2017) → normal development
	2 completed pregnancies in *JIA* (Smith and Chambers, 2018) → 2 oligohydramnios, 1 jaundice, 1 right hydrocele
	1 voluntary abortion in *unspecified rheumatological disease* (Bazzani et al., 2015)
	1 completed pregnancy in *IRP* (Youngstein et al., 2017) → normal development
	2 completed pregnancies in *TRAPS* (Youngstein et al., 2017) → normal development
	1 miscarriage in *Cogan Syndrome* (Youngstein et al., 2017)
Canakinumab human IgG kappa monoclonal antibody to IL-1Beta	5 completed pregnancies in *CAPS* (Youngstein et al., 2017; Egawa et al., 2017) → normal development
	2 completed pregnancies in *FMF* (Youngstein et al., 2017) → normal development
	1 miscarriage in *Cogan Syndrome* (Youngstein et al., 2017)
	1 completed pregnancy in *Un-SAID* (Youngstein et al., 2017) → normal development
Colchicine Alkaloid extracted from plants of the genus Colchicum (autumn crocus)	1152^a^ pregnancies in *FMF* (Ehrenfeld et al., 1987; Tutuncu et al., 2006; Ben-Chetrit et al., 2010; Bodur et al., 2020; Diav-Citrin et al., 2010; Yazicioğlu et al., 2014; Dağlar et al., 2016; Iskender et al., 2020) → 31 abortions; 14 miscarriages; 16 fetal/congenital anomalies; 8 stillbirth**;** 60 pre-term delivery; 9 SGA; 3 preeclampsia
	3 completed pregnancy in *IRP* (Brucato et al., 2019; Orabona et al., 2020) → normal development
Rilonacept Dimeric, glycosylated fusion protein composed of the extracellular domains of the interleukin 1 receptor and an accessory protein IL-1-RAcP fused to the Fc domain of human IgG1	No data

AOSD, adult-onset Still’s disease; CAPS, Cryopyrin Associated Periodic Syndrome; FMF, Familiar Mediterranean Fever; IRP, idiopathic recurrent pericarditis; JIA, Juvenile idiopathic arthritis; TRAPS, Tumor Necrosis Factor Receptor Associated Periodic Syndrome; Un-SAID, undifferentiated systemic autoinflammatory disorder.

^a^ For two studies (Bodur et al., 2020; Yazicioğlu et al., 2014), the total number of pregnancies we considered includes women that did not receive the drug therapy; for the study by Iskender et al., as the total number of pregnancies was not available, we considered the total number of pregnant women treated with the drug of interest.

## Discussion

Available data assessed from the published literature shows that the rates of major congenital deformities and miscarriages for investigated inflammasome target therapies do not differ considerably from those rates in the general population. It should be stressed that from the published evidence, despite their heterogeneousness, no new safety concerns were identified from a total of 18 reports regarding the use of inflammasomes inhibitors during pregnancy. This has been further supported by the analysis of one of the largest spontaneous reporting system databases where we systematically examined the association for all currently clinically approved IL-1 inhibitors with an enormously higher number of reports representing pregnant population (40,033), strengthening the statistical power of the analyses.

Although there is little definitive evidence for the safety of IL-1 targeted therapies in pregnancy, emerging data from prospective birth cohort studies should help inform their use in pregnancy when no other suitable alternative available that can effectively control the maternal disease. Small scale clinical studies are not robust enough to answer safety concerns. In real-world, pregnant women take drugs either intentionally or unintentionally. Intentionally because some conditions require treatment during pregnancy, whilst a large proportion of pregnancies are unplanned entails unintentional drugs (Sheffield et al., 2014). Therefore, a constant vigilance of spontaneous reporting systems is essential to guarantee the safety profile of novel or repurposed treatment options. Findings obtained from case/non-case analyses of FAERS showed no potential safety signals for maternal and fetal AEs. These results are in accordance with the emerging evidence from small scale studies assessed in the present review. In FAERS, AEs related to pregnancy and neonatal outcomes are reported more frequently for anti-TNFα than treatments targeting IL-1. This could be explained by the fact that anti-TNFα drugs are the most prescribed treatment in pregnancy because of their safety evidence has already been translated into clinical practice (Flint et al., 2016; Skorpen et al., 2016). Like any epidemiology studies, valid results from case/non-case analyses depends on the classification of events and exposure. Underreporting of ADRs due to “misclassification bias” may explain a smaller number of reports for the comparator drug in case/non-case analyses, and henceforth direction and magnitude of the effect (Carnovale et al., 2019; Mazhar et al., 2019).

Our findings expand on Nguyen et al*.* (Nguyen at al., 2020) systematic review of 11 studies that investigated the risk of adverse pregnancy and neonatal outcomes associated with non-TNFα inhibitors and disease-modifying anti-rheumatic drugs (DMARDs) in pregnancy. Their findings do not suggest an increased risk associated with non-TNF α inhibitors and DMARDs in pregnancy. Nevertheless, the methods in the two studies are different. We have investigated a range of studies focused on IL-1 inhibitors only and have summed up data on congenital malformation/miscarriages. We have also summarized the pattern of reported major congenital malformations. In the present review, each IL-1 inhibitor was considered separately that can be of additional value if one is interested in specific IL-1 inhibitor drug. Moreover, data on maternal baseline characteristics are also detailed as the underlying maternal conditions are essential factors to be considered as a potential confounder for the pregnancy-related outcomes.

According to EMA guidelines, to rule out a ≥2-fold or ≥10-fold risk of major congenital malformations for medicine use during pregnancy, there is a need of moderate amount of data on pregnant women (between 300 and 1,000 pregnancy outcomes) collected prospectively (EMEA, 2008). This information should be collected from exposed pregnancies to that particular drug during the first trimester. Under this recommendation, the ascertainment of risk on summed up data remains unclear for most of the IL-1 antagonist because of the limited total number of exposures. Although for colchicine it can be concluded that it does not carry a ≥2-fold increased risk of combined major congenital malformations as compared to this risk in the general population which was estimated as 2–5.5% (Egbe et al., 2015). Over the past few years, the approach toward the use of colchicine throughout pregnancy has changed. Older reports and the 2015 ESC Guidelines (Adler et al., 2015) recommended against the use of colchicine during pregnancy due to limited information, while available data suggest that did not raise any concern about the teratogenicity of the drug. No association between an increased rate of adverse maternal/fetal outcomes with the colchicine throughout pregnancy was found in a recent systematic review of the literature (Indraratna et al., 2018).

A recent retrospective review of 34 exposed pregnancies to colchicine (Duman et al., 2019) reported the birth of 23 healthy infants and seven with several problems. Of seven infants with problems, four had congenital heart disease, one had nephrolithiasis, one had an inguinal hernia and one had mortality secondary to respiratory distress. The investigator acknowledged that the causality between colchicine use and the reported malformations was not adequately ascertained. These findings indicate that the influence of colchicine cannot be ruled out completely and should be beard in mind in cases of colchicine use for indications other than FMF or Behcet’s disease.

Safety data on IL-1 inhibitors anakinra and canakinumab remain very limited. Reviewed real-word data appear reassuring. In a retrospective study by the International Society for Systemic Auto-inflammatory diseases, 43 pregnancies exposed to IL-1 therapies (canakinumab, n = 14 and anakinra n = 29) from seven countries (Youngstein et al., 2017), were identified. There were no developmental abnormalities with a median follow-up of 18 months. They also reported the outcome of 14 neonates breastfed by mothers taking anakinra (n = 10) or canakinumab (n = 4) for up to 10 months, with no reported serious infections.

Largely reassuring outcomes was also reported in a small case series of seven pregnant women with AOSD treated with anakinra (Fischer-Betz et al., 2011; Smith and Chambers, 2018). Of seven pregnant women, two developed oligo-hydramnios, and one developed pregnancy-induced hypertension while taking anakinra. All the infants were born full-term (range: 36–40 weeks of gestation) and there were no adverse outcomes or major complications. Of the seven mothers, three breastfed their newborns successfully, where two mothers chose to breastfeed while continuing anakinra treatment with no reported adverse outcomes. Chang et al. (2014) described 24 pregnancies in patients with CAPS, reporting a lower miscarriage rate in women on anakinra than in those treated with other drugs (10% vs. 30%) suggesting anakinra use may have a protective effect on pregnancy outcomes for patients with IL-1-driven disease. Due to the scantiness of available data on the safety of IL-1 antagonists during conception, until additional information is available, these agents are not currently recommended during pregnancy and their discontinuation should be considered once pregnancy is confirmed (Sammaritano et al., 2020).

For several reasons, it is difficult to determine the impact of medications on obstetric and neonatal outcomes. In prospective studies, early miscarriages are missed because women are only included when they are pregnant and not in the pre-conception period. Whilst retrospective studies are endangered by publication bias because only women with an abnormal pregnancy are reported (Mitchell et al., 2011; Thorpe et al., 2013). Prospective studies that enroll the women from the phase they actively try to become pregnant would cover the pre-conception phase and provide reliable data. Furthermore, confounding by indication (some diseases cause more miscarriages) and by concomitant drugs can also affect the results.

Large population-based cohort studies from routine healthcare settings are crucial for improving the evidence for the safety of IL-1 treatments during pregnancy. Use of growing number of available electronic health care databases may be valuable strategy to capture information on medicine utilization patterns, and medicine safety when used during pregnancy. However, for future non-RCT studies there is a need to overcome important methodological issues related to methods for quantifying exposures to these drugs and other confounders during the whole of pregnancy and for adjusting for the latter in analyses.

### Limitations

The robustness of uncontrolled evidence was poor in the reviewed studies. IL-1/colchicine-exposed women are likely to have other factors at some point in pregnancy as poor pregnancy outcomes are multifactorial, generally, this was not measured and so, could not be adjusted for in uncontrolled studies, making interpretation of these studies’ findings difficult. Type of disease and disease activity during pregnancy, co-morbid conditions, and concomitant drug treatments could also have contributed to negative outcomes. In some cases, the information on other outcomes (such as previous births, previous miscarriages, preterm births, ADRs, etc.) was not available. Finally, the use of a pharmacovigilance database has some intrinsic limitations; reporting might be influenced by factors including the notoriety bias, selection bias and under-reporting, which precludes making causal inferences except in unusual circumstances (Faillie 2019).

## Conclusion

Despite limitations, no major safety issues were reported and no increasing trend could be identified in the reported malformations risk of biological IL-1 therapies in pregnancy. Colchicine appears to be compatible with pregnancy. Though these provide no suggestion that biological IL-1 treatments might be harmful, however, due to limited total number of exposed pregnancies the use of these agents in the clinical practice should be considered in pregnancy only if the risk benefit assessment of maintaining disease control justifies the potential risk to the fetus. These findings contribute additional evidence to inform treatment decision making for patients with IRD during pregnancy.

## Data Availability Statement

The raw data supporting the conclusions of this article will be made available by the authors, without undue reservation.

## Author Contributions

All authors listed have made substantial, direct and intellectual contribution to the work and approved it for publication. CC, and ET conceptualized and designed the study, interpreted the data drafted the manuscript, revised and approved the final manuscript as submitted. VB participated in the conceptualization and design of the study, carried out the initial analyses, revised the manuscript and approved the final manuscript as submitted. FM, SR, MN, EN, ST participated in the interpretation of the data, revised the article, and approved the final article as submitted. AB conceptualized and designed the study, interpreted the data, coordinated and supervised data collection, critically reviewed the manuscript and approved the final manuscript as submitted.

## Funding

The Authors received financial support for the publication of this article from the Swedish Orphan Biovitrum (Sobi™).

## Conflict of Interest

The authors declare that the research was conducted in the absence of any commercial or financial relationships that could be construed as a potential conflict of interest.
