# Supplementary material for: Inflammasome Targeted Therapy in Pregnancy: New Insights From an Analysis of Real-World Data From the FAERS Database and a Systematic Review
Source: Front Pharmacol. 2021 Jan 20;11:612259. doi: 10.3389/fphar.2020.612259 (PMC7854464; doi:10.3389/fphar.2020.612259)
Supplement: Supplementary file 1 [file datasheet1.docx]

**Supplementary material 1.** Electronic search strategy for PubMed database.

("Interleukin 1 Receptor Antagonist Protein"[Mesh] OR "Colchicine"[Mesh] OR "IL1 Febrile Inhibitor" [Tiab] OR "Urine-Derived IL1 Inhibitor"[Tiab] OR "Urine Derived IL1 Inhibitor"[Tiab] OR "IL-1 Inhibitor"[Tiab] OR "IL 1 Inhibitor"[Tiab] OR "Interleukin 1 Inhibitor"[Tiab] OR "Antril"[Tiab] OR "Kineret"[Tiab] OR "Anakinra"[Tiab] OR "Canakinumab"[Tiab] OR "Rilonacept"[Tiab] OR "Colchicine"[Tiab]) AND ("Pregnancy"[Mesh] OR "Prenatal Care"[Mesh] OR "Maternal-Fetal Relations"[Mesh] OR "Pregnant Women"[Mesh] OR "Maternal Fetal" [Tiab] OR "Mother-Fetus" [Tiab] OR "Mother Fetus" [Tiab] OR "Pregnancy" [Tiab] OR "Pregnant" [Tiab] OR "Pregnancies" [Tiab] OR "Gestation" [Tiab] OR "Antenatal Care" [Tiab] OR "Foetus" [Tiab])
